# The effect of increased unplanned surgical admissions at a 12 bed critical care unit

**DOI:** 10.1186/2197-425X-3-S1-A534

**Published:** 2015-10-01

**Authors:** NT Nelligan, A Claxton, D Dutta

**Affiliations:** The Royal London Hospital, Barts Health Trust, London, United Kingdom; The Princess Alexandra Hospital Trust, Anaesthetics and Critical Care, Harlow, United Kingdom; South Warwickshire Foundation Trust, Warwick, United Kingdom

## Introduction

Unplanned surgical admissions to critical care are indicative of poor outcome in surgical patients [1].There has been an increase in the number of unplanned admissions to critical care units, which impacts on the allocation of resources, affecting mortality [2].The hospital where this study took place has seen a doubling of admissions in the last 5 years [3].A large proportion appeared to be surgically related, prompting a review if outcomes have been jeopardised by the increased strain.

## Objectives

To establish a trend in admissions by month, over 6 months for unplanned surgical admissions to a London fringe hospital & to determine:

· Mortality rates for the unplanned admissions; compare to previous figures in 2008 and the Intensive Care National Audit & Research Centre (ICNARC) 2012 report.

· Mortality while on critical care, mortality from discharge to ward & discharge from hospital.

· Length of stay & APACHE II scores by outcome for unplanned surgical admissions.

· Volume of patients by speciality & survival to discharge from hospital by surgical speciality.

## Methods

This is a retrospective analysis of 142 unplanned surgical admissions over 6 months, from 1^st^ July-31^st^ December 2013, in a London fringe hospital with a 12 bed critical care unit.

A database was developed on excel using coded data & contained the breakdown of each admission, expected outcome predicted by APACHE II score, outcomes including mortality on ITU/HDU, mortality after discharge to ward & the length of stay by outcome for each of the admissions. The ICNARC 2012 data on similar sized units was analysed & the 2008 study from the same trust reviewed. We compared to ICNARC data & the local results from 2008 to assess how the unit is performing regarding patient outcomes & to see what, if any, changes had occurred since 2008.

## Results

## Conclusions

Despite an increased demand, the overall outcomes compare favourably with national standards, & previous figures from 2008. Currently patient outcome is not jeopardised by the increased demand on the service. However, if the trend of increasing critical care admissions (planned or unplanned) is to continue at this rate the evidence suggests that good outcomes are unlikely to remain [2].The study also reveals the largest contributing speciality to unplanned admissions was general surgery & we noted a curious trending of admissions in October/November, which warrants further analysis.

**Figure 1:**
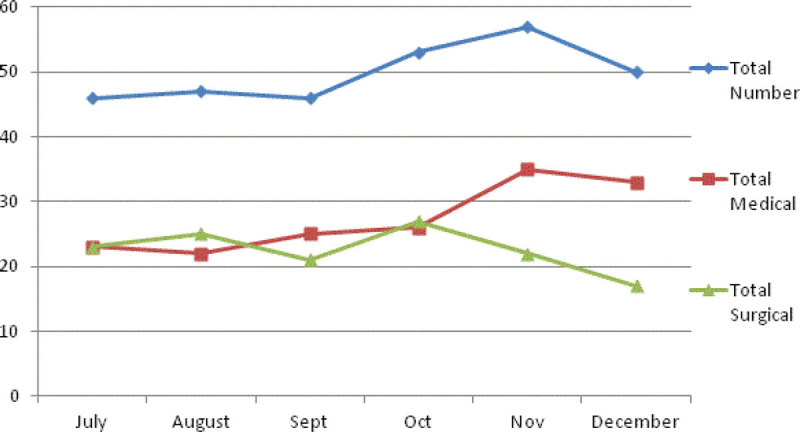
***Trend of admission numbers for study period.***

**Figure 2:**
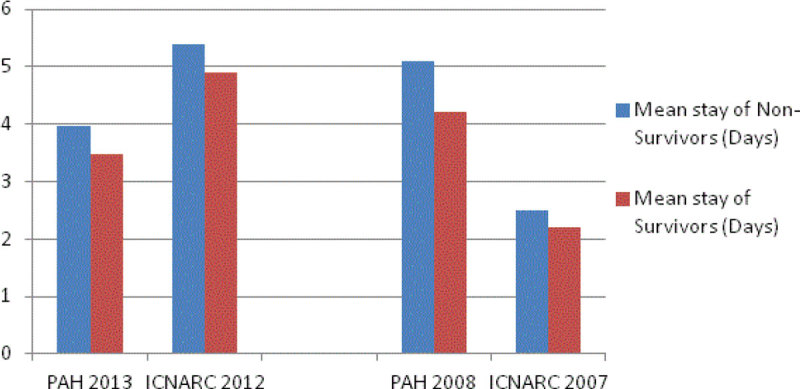
***Length of stay by Outcome compared to ICNARC 2012.***

**Figure 3:**
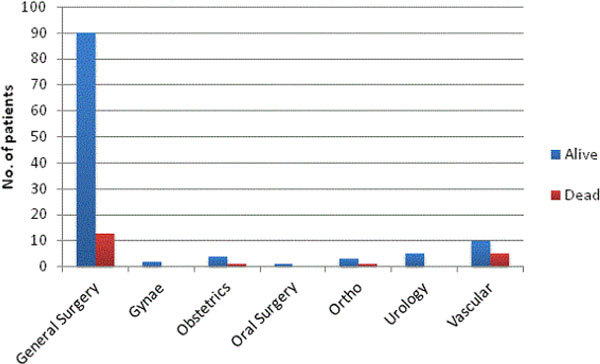
***Volume of patients & Outcome at hospital discharge.***

**Table 1:** *APACHE II scores.*

MEAN APACHE II	15
MODE APACHE II	17
RANGE OF VALUES	2 - 43
Expected Mortality based on Mean	25%

**Table 2:** *Unplanned surgical admissions Mortality Rate (%).*

Mortality rate 1st July-31st Dec 2013	8.89%
ICNARC 2012 mortality	11.9%

**Table 3:** *% unplanned admissions compared to 2008 & ICNARC.*

% of total unplanned admissions that were surgical (In study period)	45%
% of total unplanned admissions that were surgical (2008 data)	26%
National figures - ICNARC 2012	17.8%
